# Gunshot Wound

**Published:** 1865-12

**Authors:** 


### GUN-SHOT WOUND.

Dr. G. A. Wunsick, late Assistant Surgeon U. S. Vols., reports in the New York *Medical Journal* a case of gun-shot injury of the median and internal cutaneous nerves of the right arm, which was treated unsuccessfully by dissection of the cicatrix from the nerves, and excision of a portion of them, together with subcutaneous injections of morphia.

The patient, a soldier, suffered intense pain, symptoms of trismus supervened, and amputation was resorted to eventually and the patient recovered.

[Several cases somewhat similar to this one came under our observation during the war, in some of which the ordinary means failed to afford relief. In the case of a soldier in a field hospital near Murfreesboro', Tenn., in which the injury was of the nerves of the leg, the only relief he had was when the part was immersed in cold water.] L.

NOTICE.—The Dissecting Room of Rush Medical College will be open during the months of February, March and April, 1866, under the supervision of Drs. Powell and Lackey, for the study of Practical Anatomy and Surgical Operations. Special attention will be given to the demonstration of the Nervous and Venous Systems.

